# Sertraline-Induced Sleep Paralysis: A Case Report

**DOI:** 10.7759/cureus.49014

**Published:** 2023-11-18

**Authors:** Maninder Sohi, Lakshit Jain, Michael Ang-Rabanes, Raja Mogallapu

**Affiliations:** 1 Psychiatry, West Virginia University School of Medicine, Martinsburg, USA; 2 Psychiatry, University of Connecticut, Hartford, USA

**Keywords:** rem sleep, mdd, major depressive disorder, serotonin, ssris, depression, parasomnias, sleep paralysis

## Abstract

Major depressive disorder (MDD) is associated with both insomnia and hypersomnia, but it predominantly decreases sleep continuity and leads to a decrease in rapid eye movement (REM) latency, an increase in REM sleep duration, and an increase in REM density. Some of these changes persist even when MDD is treated and can be associated with a recurrence of MDD. Antidepressants can potentially complicate the relationship between REM sleep and depression, as a majority of patients report improved sleep when prescribed selective serotonin reuptake inhibitors (SSRIs) but some case reports mention that SSRIs have been associated with REM inhibition, resulting in decreased REM sleep.

We present a case report of a young patient with MDD who started experiencing multiple episodes of distressing sleep paralysis after he started taking sertraline and resolved as he was tapered off the medication.

Through references from the literature indicating a potential link between parasomnias and SSRIs, we were able to discuss that SSRIs can potentially lead to isolated sleep paralysis and should be considered as an uncommon yet distressing side effect although not listed in the package insert. Isolated sleep paralysis has been defined in the literature as the inability to perform voluntary movements of the trunk and all limbs for a period of seconds to minutes at the beginning of sleep or upon waking up.

Further research is needed to clarify the impact of SSRIs on sleep and practice guidelines should be clarified in regard to their role.

## Introduction

Depression and sleep have a historical, close connection and can impact each other [1]. Major depressive disorder (MDD) is a complex disorder that is associated with a heterogeneous patient presentation and there is likely more than one underlying mechanism and pathway behind this [2]. MDD is associated with both insomnia and hypersomnia, but in many cases, it may decrease sleep continuity and can be associated with a decrease in rapid eye movement (REM) latency (time between sleep onset and the first REM sleep period), an increase in REM sleep duration, and an increase in REM density along with decreased slow-wave sleep [3,4]. Some of these polysomnographic changes can persist even when depression is treated and can be associated with the recurrence of depression or other psychiatric disorders, highlighting the role REM sleep plays in cognition and emotion [3].

While antidepressants generally improve sleep, they may also disrupt the relationship between REM sleep and depression. Although the majority of patients note improvement in sleep when prescribed SSRIs, some reports mention that SSRIs have been associated with REM inhibition, resulting in decreased REM sleep duration and increased REM latency [3]. They also lead to sleep fragmentation through the inhibition of “REM-on” neurons [4]. In REM sleep, the brain is “awake” with vivid dreaming, leading to rapid eye movement; and the body is “paralyzed” with low muscle tone. Persistence of REM atonia can lead to sleep paralysis, which is commonly benign in nature, but it can be a terrifying phenomenon as the patient reports a transient inability to speak or move the trunk or limbs during sleep-wake transitions [5,6]. It is often seen as part of the symptoms of narcolepsy, but when it occurs by itself, it is termed isolated sleep paralysis (ISP), and if there are multiple episodes, it is termed recurrent isolated sleep paralysis (RISP) [5,6].

RISP is estimated to affect around 8% of the general population according to some studies; however, the reported prevalence of sleep paralysis varies widely, as the literature notes that approximately 15% to 40% of younger individuals experience at least one episode of sleep paralysis [5-7]. ISP can also be accompanied by vivid hallucinations [5,6]. The diagnostic criteria for RISP include episodes lasting seconds to minutes and causing clinically significant distress to the patient. One must also rule out other sleep disorders such as narcolepsy, psychiatric disorders or medical conditions, medication use, or substance use disorders [6]. A further way some have described this diagnosis in the literature is to term it fearful ISP, as these sleep paralysis episodes can cause clinically significant fear or distress to the patient [7]. Some researchers propose that sleep paralysis can be treated with antidepressants [8]; sertraline is a commonly prescribed medication for depression. However, it is important to be aware that it can cause sleep paralysis, which can be a distressing experience. Our case presentation sheds light on this issue and highlights the risks associated with the use of sertraline.

## Case presentation

A 26-year-old male with no prior medical conditions presented to the Crisis Response Centre (CRC) complaining of terrifying visual hallucinations. He reported that he was diagnosed with MDD recently and was taking sertraline 100 mg daily for the past three months at the time of presentation. The patient stated that his depressive symptoms improved with the medication. He reported experiencing good sleep initially, but later, he began having episodes of emerging from sleep to a state of being fully awake but unable to move as if his body was “locked.” Typically, these episodes would occur from a nocturnal awakening, and the entire episode lasted for approximately 10 minutes. This was accompanied by a sense of intense fear and breathlessness. He also reported experiencing a “strange sensation,” feeling a distinct foreign presence in the room, seeing shadows, and hearing footsteps during this episode. As soon as he felt his body was “unlocked," he found himself drenched in sweat. He reported that in the last few days, the episodes were increasing in frequency, with the last episode being one day prior to his arrival at the CRC although these episodes initially began two weeks prior to presentation. These were so distressing for the patient that he skipped work and did not sleep for 36 hours, as he was fearful of experiencing this incident again and preoccupied with ways to avoid it. The patient had no prior history of parasomnia. He denied restlessness in his legs, snoring, witnessed apnea, and cataplexy. He stated that he was very afraid and might “kill himself” if he had a similar episode, which led to him being admitted to the inpatient psychiatric unit.

In the inpatient unit, his initial admission assessment was negative for symptoms of any medical illness, and his admission labs (complete blood count, comprehensive metabolic panel, urine drug screen, computed tomography scan of the head, and EKG) were unremarkable. As he reported that his symptoms began after he started taking sertraline for his MDD, sertraline was tapered off in the next few days. This was associated with a decrease in the intensity and frequency of parasomnia episodes, which added to our suspicion. With the decrease in dose, his depressive symptoms returned, and the treatment team started him on bupropion 75 mg. The patient responded well to this regimen and slowly the bupropion was titrated up to 300 mg with a significant reduction of his depressive symptoms and suicidal thoughts. He reported having trouble falling asleep, so trazodone 50 mg was added to his regimen. The patient reported complete resolution of parasomnia episodes and was discharged on this regime with advice to follow up as an outpatient.

## Discussion

We were fortunate, as the patient was a young man with no medical or other psychiatric comorbidities, which helped us quickly identify the cause of his distress. The likelihood that these episodes of ISP were the result of substance use is low due to the distressing nature of these episodes, lack of history of substance use, and the fact that the urine drug screen was negative on admission. However, the possibility that the patient could be withholding information from us cannot be ruled out. An A-B-A design (on sertraline, off sertraline, and then on sertraline) would have been helpful for establishing a relationship between sertraline and sleep paralysis, and we could have potentially performed a trial of sertraline during his inpatient stay to confirm that sertraline was indeed leading to these symptoms. Unfortunately, due to the distressing nature of these symptoms, the patient declined this retrial of sertraline. A polysomnography study could also have been useful in supporting our diagnosis of ISP, but we were not able to perform it in this patient due to the short duration of the inpatient stay.

SSRI treatment of depression commonly results in improved sleep symptoms and subsequent improvement in mood [4]. However, serotonin can enhance the wake state by inhibiting REM sleep [4,9], and there are some case reports linking parasomnias with SSRIs [3,4]. Just like in our case, a non-SSRI antidepressant, such as bupropion, can be a viable treatment option. Bupropion has additional utility, as it reduces REM sleep to a lesser extent than other antidepressants, leading to prolonged REM sleep latency and increased REM density [4].

Like us, many clinicians utilize trazodone in the treatment plan to offset some of the undesirable sleep-related changes from SSRI monotherapy [4]. While high-dose trazodone can provide some SSRI properties, it acts as a 5-HT2A receptor antagonist at low doses with potent α1-adrenergic antagonism causing sedation [4]. Providers should also consider discussing sleep hygiene to prevent sleep deprivation, and REM-suppressing agents like benzodiazepines can be utilized based on the degree of patient distress [5,10].

Although the mechanism behind REM motor atonia has not been identified, the literature implies multiple possible mechanisms, which involve both metabotropic gamma amino butyric acid (GABA) GABAB and ionotropic GABAA/glycine receptor-mediated inhibition of skeletal motor neurons as the underlying mechanism of REM motor atonia [11,12]. Future research in this direction can lead to new medications to provide treatment that is more effective. Finally, this case report highlights the importance of considering parasomnias as potential side effects of SSRIs and informing the patient of this side effect when prescribing them in addition to asking about changes related to sleep at clinic follow-up as this can cause significant distress.

Current practice guidelines appear to be lacking in regard to managing ISP. It is important to rule out narcolepsy, obstructive sleep apnea, migraine headaches, stroke, anxiety disorders, and other REM sleep disorders before making a diagnosis of ISP.

## Conclusions

It is imperative that practitioners remain vigilant regarding the possibility of sleep paralysis as a rare yet consequential side effect when prescribing antidepressants to treat patients with depression. Antidepressants have been correlated with both NREM and REM parasomnias, making it crucial to maintain a high level of suspicion while prescribing these medications and when patients voice concerns about their sleep.

Treatment options for ISP are limited, but practitioners should start with providing reassurance, education about sleep hygiene, and monitoring for sleep deprivation by taking a detailed history. One must enquire for concurrent use of antidepressants in a patient suffering from ISP, and if the patient is on antidepressants, especially SSRI and SNRIs, consideration should be given to utilizing alternatives such as bupropion, trazodone, mirtazapine, and/or low-dose benzodiazepines.
